# A novel heuristic Morlet wavelet neural network design for the painlevé equation-II arising in nonlinear optics

**DOI:** 10.1038/s41598-025-10096-w

**Published:** 2025-07-09

**Authors:** Sundas Faisal, Zulqurnain Sabir, Samra Urooj Khan, Muhammad Aamir, Krzysztof A. Cyran

**Affiliations:** 1 Department of Graphics, Computer Vision and Digital Systems, Silesian University of Technology, Gliwice, Poland; 2 Department of Computer Science and Mathematics, Lebanese American University, Beirut, Lebanon; 3 Faculty of Electrical and Electronic Engineering, Universiti Malaysia Pahang Al-Sultan Abdullah, Pekan, Pahang, 26600 Malaysia; 4 Department of Computer Science, University of Oxford, Wolfson building, Parks Road, Oxford, OX1 3QG UK

**Keywords:** Painlevé equation-II, Nonlinear optics, Morlet wavelet, Neural networks, Genetic algorithm, Interior-point algorithm, Mathematics and computing, Optics and photonics

## Abstract

The current investigations present the novel structure of Morlet wavelet neural network (MWNN) for the numerical solutions of Painlevé equation-II arising in nonlinear optics. An error based fitness function is constructed using the sense of differential model and its initial or boundary conditions, which is further optimized through the hybrid computing terminologies of the global search genetic algorithm (GA) and local search interior-point algorithm (IPA), i.e., GA-IPA. The precision of the process is observed through the overlapping of the proposed and reference results, and negligible absolute error. Moreover, the statistical analysis using the multiple independent runs is performed by different tests including Theil’s inequality coefficient, variance account for and semi inter-quartile range, which shows the dependability of the proposed MWNN-GA-IPA in order to Painlevé equation-II arising in nonlinear optics. The proposed MWNN-GA-IPA optics is discussed for the first time for solving the Painlevé equation-II arising in nonlinear.

## Introduction

Paul Painlevé derived all types of Painlevé equations (PEqs) in the early 20th century, and later one of his students Gambier derived a cataloging of such equations in the form of $z^{\prime\prime}=g(x,z,z^{\prime})$, where *g* represents a function of *z* and its 1st order derivative and meromorphic in *x*, which obey the Painlevé property and have no moveable singularities of the results with current poles^1^. PEqs represent one of the categories of nonlinear ordinary differential systems, which have been widely investigated in physics, mathematics and engineering, like as theory of random matrix, orthogonal polynomials, Frobenius manifolds, reduced integro partial differential systems, number theory, enumerative algebraic geometry, and dynamical systems^2,3^. There are six PEqs (P-I to P-VI) or Painlevé transcendent, which become essential objects based on investigations of integrable models to exhibit different properties, e.g., algebraic-geometric constructions, Lax pairs, and soliton solutions^4^. It is not possible to reduce Painlevé transcendent to standard unique capabilities, however, the classical solutions of PEqs are presented in the literature using the special parameters, e.g., airy, hypergeometric, Bessel and Hermite functions^5^. In mathematics, PEqs are linked to differential and algebraic geometry, while they play a vital role to present the critical phenomena in the area of physics, e.g., quantum field theory and phase transitions^6^.

These equations have been implemented to model the real-world systems, like population/fluid dynamics, and nonlinear optics. In nonlinear optics, PEqs describe the optical pulses conduct and beams in numerous media^7^. Generally, P-II and P-III equations provide the spread of ultra-short pulses of optical fibers, whereas P- IV and P-V are used to model the spatial solitons using the nonlinear waveguides. These equations are used to capture the impacts of cross-phase modulation, self-phase inflection, and dispersion, which allowing the investigation of complex phenomena, e.g., soliton formation, optical shock waves, and pulse density^8,9^. Moreover, P-VI has been associated to investigate the beam propagation and optical vortex solitons in nonlinear media along with the index of variable refractive. To solve these equations, the scholars can optimize and design the system of optical communication, lasers, and amplifiers, for controlling and predicting the nonlinear impacts in photonic plans^10,11^. The motive of current research investigations is to provide the numerical solutions of P-II, which arise in the studies of nonlinear optics, and mathematically written as^12^:

1 $$\left\{ \begin{gathered} \frac{{{d^2}y(x)}}{{d{x^2}}}=2{y^3}(x)+xy(x)+\lambda , \hfill \\ y(0)=\frac{{dy(0)}}{{dx}}=0. \hfill \\ \end{gathered} \right.$$

The PEq shown in system (1) arises in the nonlinear optics, which transmute the complex amplitude based on the propagation of electric field in an optical fiber using the exact non-stationary results of the renowned nonlinear Schrödinger (NS) models. These solutions are signified through P-II by using the suitable transformations. The optical pulses transmission is stated with the NS model is given as^13^:

2 $${U_x}=j\left( { \pm {U_{tt}} - 2U{{\left| U \right|}^2}} \right),$$

where the complex electrical field amplitude is signified by *U*(*x*, *t*), which is shown as:

3 $$\eta =t+x(g+hx),$$

where $\varphi (x,t)$ and $R(x,t)$ are the phase and amplitude of the complex number. The anticipated results by applying the below transformation are presented as:

4 $$U(x,t)=\operatorname{R} (x,t){e^{\left( {j\varphi (x,t)} \right)}},$$

while the phase $\varphi (x,t)$ is shown as:

5 $$\phi =ax+bt+cxt+d{x^2}+e{x^3}.$$

where *a*, *b*, *c*, *d*, *e*, *g*, and *h* represent the free parameters, while the precise values of these parameters are given as:

6 $$b= \mp 0.5g,c= \mp h,d= \mp 2gh,e= \mp \frac{2}{3}{h^3}$$

The above Eq. (3) to (5) are used in (6), the updated equation for *R* becomes as:

7 $$\mp \frac{{{d^2}R}}{{d\eta }}=R\left( {a+0.25{g^2} \mp h\eta } \right)+2{h^3}.$$

For *h* = 0, the system (2) produces the stationary result as:

8 $$x=\frac{{\left( {0.25{g^2} \mp a} \right)}}{h},\,{p^3}= - \frac{1}{h},{A^2}={h^{2/3}}.$$

The family of the results for *y* using the P-II is drawn by putting *λ* = 0 as:

9 $$\frac{{{d^2}y(x)}}{{d{x^2}}}=xy(x) \pm 2{x^3}.$$

The cubic factor with positive sign is taken with the velocity dispersion. The equation based P-II has widely been investigated by many scholars in various schemes^14,15^. A thorough review of the literature reveals that the P-II arising in nonlinear optics cannot yet be numerically solved by using the Morlet wavelet neural network (MWNN) enhanced by the optimization of global search genetic algorithm (GA) and local search interior-point algorithm (IPA), i.e., GA-IPA. This was one of the research gaps and the authors of this study are interested to fulfil this gap by using the MWNN-GA-IPA for solving the P-II arising in nonlinear optics. However, the stochastic solvers have been exploited in the number of applications in current years, some of them are food chain models^16^, Zika system-based reservoirs and human movement^17^, nonlinear singular models of Lane-Emden types^18^, economic and environmental mathematical system^19^ and nonlinear infectious disease model considering its anatomical variables^20^. Some novel features of this study are presented as:

- A differential P-II arising in nonlinear optics is solved competently by using the stochastic computing paradigms.
- The design of MWNN is explored for the first time to solve the P-II arising in nonlinear optics.
- The optimization is accomplished through the hybridization of global and local search operators, named as GA-IPA.
- A fitness function is considered by the differential P-II arising in nonlinear optics, which is further optimized by the combination of GA-IPA.
- The correctness is observed through the comparison of the reference and obtained solutions.
- The statistical performances are also provided in order to observe the accuracy of the proposed solver.

The rest parts of the paper are summarized as: Sect. 2 shows the mathematical modelling of the MWNN-GA-IPA, Sect. 3 shows the numerical results and discussions, while the conclusions are reported in the final section.

## Methodology

This section presents a novel design of the MWNN under the optimization of GA-IPA for presenting the numerical solutions. The modeling is based on two phases as:

- A fitness function is constructed using the mathematical P-II arising in nonlinear optics model.
- The optimization of fitness function is performed through the hybridization GA-IPA.

### Construction of the MWNN

In this study, the activation Morlet wavelet kernels (MWKs) along with the optimization of GA-IPA is used as an alternative of tanh activation or log-sigmoid windowing functions, which allow the system to study complex designs and relations in signals. MWNN is one of the kinds of feed-forward neural network, which incorporates MWKs into its construction. This function shows an outstanding time-frequency localization, and continuous wavelet transmute, which assist the system to capture both temporal and spatial designs. The structure of MWNN has been effectively functional to solve a number of tasks, e.g., time-series prediction, signal dispensation, and image compression. Some of the major advantages of MWNN are decrease requirements of the training, improved noise strength, and enhanced feature extraction. The mathematical construction of MWNN for solving the P-II arising in nonlinear optics is presented as:

10 $$\hat {y}(x)=\sum\limits_{{i=\,1}}^{r} {{k_i}Q({w_i}x+{j_i})} ,\frac{{{d^{(n)}}}}{{d{x^{(n)}}}}\hat {y}(x)=\sum\limits_{{i=\,1}}^{r} {{k_i}\frac{{{d^{(n)}}}}{{d{x^{(n)}}}}Q({w_i}x+{j_i})}$$

In the system (10), *r* shows the neurons, the unidentified weight vector is ${\varvec{W}}=[{\varvec{k}},{\varvec{w}},{\varvec{j}}]$, i.e., ${\varvec{k}}=[{k_1},{k_2},...,{k_r}],\,{\varvec{w}}=[{w_1},{w_2},...,{w_r}]\,\,and\,\,{\varvec{j}}=[{j_1},{j_2},...,{j_r}].$ For the solutions of the model, the MWNN is never been exploited before, which is mathematically given as:

11 $$Q(x)=\cos \left( {\frac{4}{3}x} \right){e^{\left( { - \frac{1}{2}{x^2}} \right)}}$$

The updated form of Eq. (10) is shown as:

12 $$\begin{gathered} \hat {y}(x)=\sum\limits_{{i=1}}^{r} {{k_i}\cos \left( {\frac{4}{3}({w_i}x+{j_i})} \right){e^{ - \frac{1}{2}{{({w_i}x+{j_i})}^2}}}} , \hfill \\ \frac{d}{{dx}}\hat {y}(x)=\sum\limits_{{i=1}}^{r} { - {k_i}{w_i}{e^{ - \frac{1}{2}{{({w_i}x+{j_i})}^2}}}\left( {\sin \left\{ {\frac{4}{3}({w_i}x+{j_i})} \right\}+\frac{4}{3}({w_i}x+{j_i})\cos \left\{ {\frac{4}{3}({w_i}x+{j_i})} \right\}} \right)} , \hfill \\ \frac{{{d^2}}}{{d{x^2}}}\hat {y}(x)=\sum\limits_{{i=1}}^{r} { - {k_i}w_{i}^{2}{e^{ - \frac{1}{2}{{({w_i}x+{j_i})}^2}}}\left( \begin{gathered} 3.0625\cos \left\{ {\frac{4}{3}({w_i}x+{j_i})} \right\} \hfill \\ +\frac{7}{2}({w_i}x+{j_i})\sin \left\{ {\frac{4}{3}({w_i}x+{j_i})} \right\} \hfill \\ +\left\{ { - 1+{{({w_i}x+{j_i})}^2}} \right\}\cos \left\{ {\frac{4}{3}({w_i}x+{j_i})} \right\} \hfill \\ \end{gathered} \right)} , \hfill \\ \end{gathered}$$

A fitness function ${E_F}$ is shown as:

13 $${E_F}={E_{F - 1}}+{E_{F - 2}}$$

where ${E_{F - 1}}$ and ${E_{F - 2}}$ represent the unsupervised error based differential system and its corresponding initial conditions, which is given as:

14 $${E_{F - 1}}=\frac{1}{N}\sum\limits_{{i=1}}^{r} {\left( {\frac{{{d^2}}}{{dx^2}}{{\hat {y}}_i} - 2\hat {y}_{i}^{3} - {x_i}{{\hat {y}}_i} - \lambda } \right)} ,\,\,\,0 \leqslant {x_i} \leqslant 1,$$

and

15 $${E_{F - 2}}=\frac{1}{2}{\left( {{{\hat {y}}_0}} \right)^2}+\frac{1}{2}{\left( {\frac{d}{{dx}}{{\hat {y}}_N}} \right)^2}.$$

### Optimization through GA-IPA

In this section, the optimization through GA-IPA is presented for solving the model. The detail of the optimization based global GA and local search IPA is presented as:

*GA* is one of the global search optimization based procedure used to solve the P-II arising in nonlinear optics model in this study, which ensure the accurate predictions. It is a consistent global search scheme, which is implemented to unconstrained, and nonlinear systems using its significant operators called mutation, elitism, selection, and crossover. The global search GA’s competence allow the consideration of the whole parameter space, justifying the local optima confines. This scheme is used to initiate a population based on 100 arbitrarily generated solutions that develop more than 500 generations by using the mutation, crossover, and selection operators. The fitness function using the mean square error (MSE) directed the search to optimal parameter groupings. The selection of tournament and elitism preserved the optimal results, whereas adaptive rate of mutation preserved diversity.

The global search GA produced extraordinary developments in the system performance, with negligible MSE in comparison with the local search schemes. These values of the optimized parameters represent the notable robustness based on the diverse situations, e.g., changeable animal densities, intervention policies, and environmental conditions. In current decades, GA has been used in a number of applications, such as chest X-ray images^21^, energy consumption in an office building^22^, supply chain finance under information sharing^23^, housing load shifting to reduce the bill reduction^24^, path autonomous UAV preparation to target the coverage systems^25^, and robust space and time gathering line balancing in uncertain petition^26^. These potential submissions motivated the scholars to apply the process of global search GA for accomplishing the decision variables of MWNN-GA-IPA to solve the model.

The local search IPA is applied to present the solutions of the model. This system designates the performance of optical pulses in fibers, which is categorized by a nonlinear factor that shows the soliton formation. IPA is one of the significant tools to solve the problems based on the constrained optimization and adapted to present the numerical performances of the P-II. The scheme iteratively upgrades the results by reducing the residual between the present approximation and the exact results associated to limitations, which certify the solution fulfils the differential equations. IPA is executed by using the discretization of finite difference for the P-II that indicate the outcomes of a nonlinear algebraic system. The efficiency of the scheme is improved by manipulating the sparsity based on the Jacobian matrix, which apply a solver of preconditioned conjugate gradient. Numerical tests represent the competence and accuracy of the solver for the P-II, with outcomes indicate the outstanding arrangement using analytical results as well as other numerical schemes. IPA indicates an efficient and reliable tool to simulate the nonlinear optical sensations and has potential submissions in associated fields, like fluid dynamics and plasma physics. Recently, IPA is used in frequent applications, some of them are image processing^27^, multistage nonlinear non-convex programs^28^, bio-convection nanofluid flow via stretching surface^29^, optimal coordination of automated vehicles at intersections^30^, and class of hypoelastic-plastic models with memory surface^31^.

### Limitation of MWNN

**Fig. 1:**
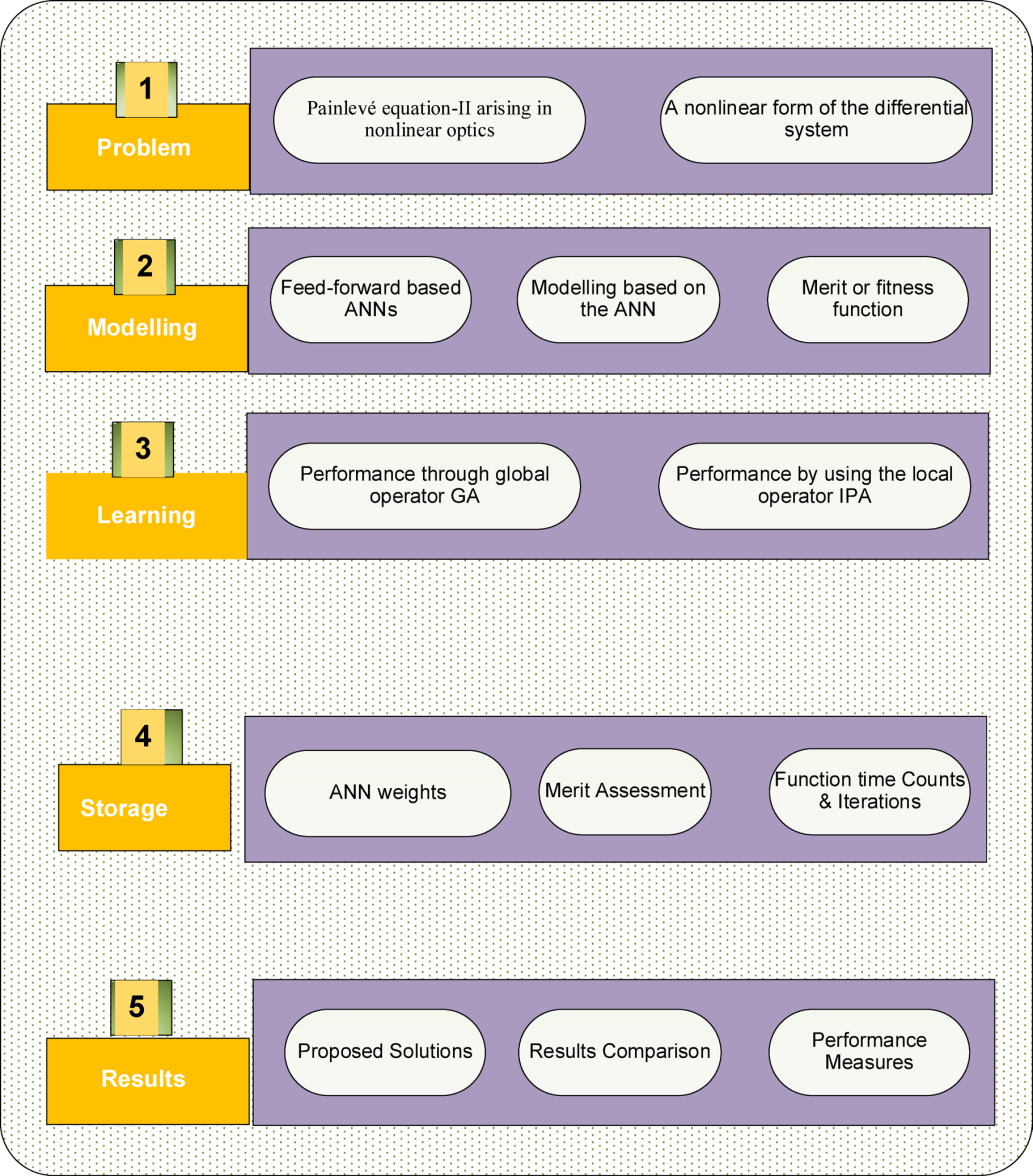
MWNN-GA-IPA structure for the P-II arising in nonlinear optics model.

The algorithm based on MWNN has performed various limitations including computational complexity, which may perform resource-intensive and time-consuming to train, mainly for complex systems or large data. In addition, the performance of the scheme can be complex to selection of parameter, e.g., selection of wavelet translation and scale parameters, which involves cautious tuning to accomplish optimal outcomes. Additionally, some other neural network schemes, MWNN may suffer through overfitting, particularly when the dataset based training is noisy or small. One more limitation is the inadequate result interpretability of the outcomes, which perform challenging to comprehend the fundamental relations and dynamics apprehended by the system. The use of the scheme based wavelet transmutes and neural networks may require cautious weights initialization and parameters to certify best performance and convergence. The structure based on MWNN-GA-IPA for the P-II arising in nonlinear optics model is presented in Fig. 1 and the process of hybridization to control the slowness of GA with IPA through the practice of optimization is shown in Table 1.

**Table 1:**
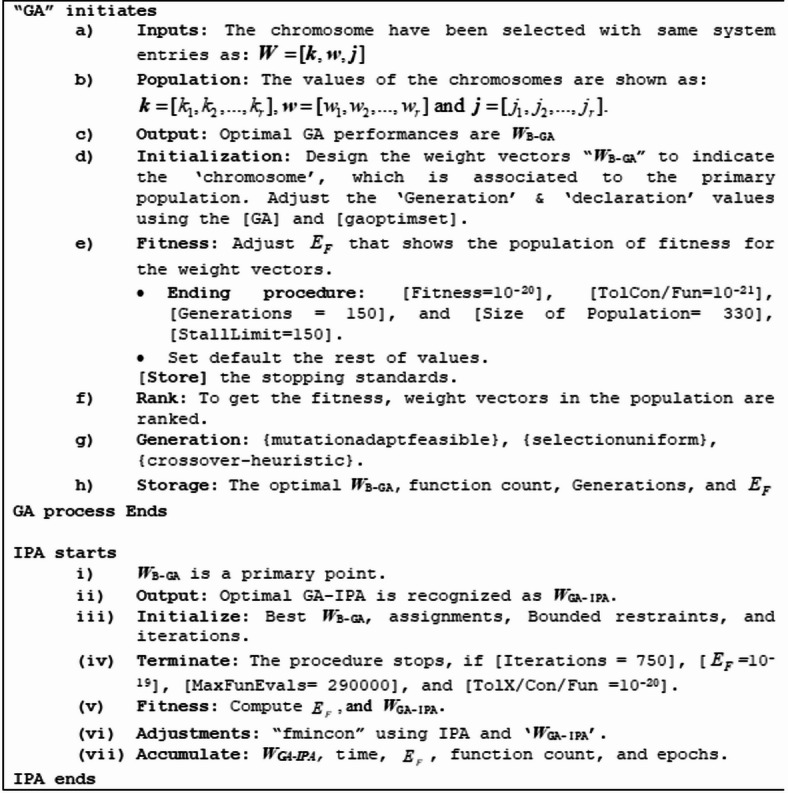
Optimization through MWNN-GA-IPA presented in pseudo code for the P-II arising in nonlinear optics model.

## Statistical measures

The statistical values based on Nash Sutcliffe Efficiency (NSE), Theil’s Inequality Coefficients (TIC), Semi-Interquartile Range (SIR), and Mean Absolute Deviation (MAD). NSE is one of the statistical metric applied to perform the projecting hydrological systems. The range of NSE is -∞ to 1. The values around 1 showing better performance of system, and shows perfect fitness in observed and calculated performances. The values lesser than zero present the mean of the experiential data is an improved predictor than the system.

TIC is another statistical performance applied to assess the precision of predictive systems. It measures the difference in predicted and observed performances having range of 0 to 1, where 1 shows the poorest possible fitness and 0 represents a perfect fitness. TIC evaluates the ratio of prediction error associated to the total data variability. A small value of TIC shows the performance in a better way having close to zero values present more precise calculations. TIC is helpful to compare different model performances or predicting schemes, which are helpful to recognize which deliver most consistent predictions.

SIR is one of the statistical amount of dispersion or variability, which is performed as ½ the difference in the 3rd quartile (*q*_3_) and 1st quartile (*q*_1_). It shows into the banquet of the middle data of 50%, which deliver an efficient variability performance less profound to outliers in comparison with the standard deviation (SD).

MAD is also one of the statistical performances, which performs the absolute average difference in separate data points and the median (Med) or data mean. It delivers a ration of dispersion or variability, which present the data spread out through the central propensity. It is measured through adding the absolute differences in each point of the data and Med or mean, then separating by the number of clarifications. The small values of MAD represent that the points of data are nearer to mean, signifying less inconsistency, whereas a higher performance of MAD represents greater spreading. MAD is an intuitive or robust amount of variability, which is widely applied in quality control, forecasting, and analysis of data.

The mathematical form of NSE, TIC, SIR and MAD are shown as:

16 $$\left\{ \begin{gathered} {\text{NSE=}}1 - \frac{{{{\sum\limits_{{i=1}}^{s} {\left( {{y_i} - {{\hat {y}}_i}} \right)} }^2}}}{{{{\sum\limits_{{i=1}}^{s} {\left( {{{\hat {y}}_i} - {{\bar {y}}_i}} \right)} }^2}}},{{\bar {y}}_i}=\frac{1}{s}\sum\limits_{{i=1}}^{s} {{y_i},} \hfill \\ {\text{ENSE=1}} - {\text{NSE}}{\text{.}} \hfill \\ \end{gathered} \right.$$

17 $$TIC{\text{=}}\frac{{\sqrt {\frac{1}{s}{{\sum\limits_{{i=1}}^{s} {\left( {{y_i} - {{\hat {y}}_i}} \right)} }^2}} }}{{\left( {\sqrt {\frac{1}{s}\sum\limits_{{i=1}}^{s} {y_{i}^{2}} } +\sqrt {\frac{1}{n}\sum\limits_{{i=1}}^{s} {\hat {y}_{i}^{2}} } } \right)}},$$

18 $$\left\{ {{\text{SIR=}} - \frac{1}{2} \times \left( {{q_1} - {q_3}} \right).} \right.$$

19 $${\text{MAD=}}\sum\limits_{{i=1}}^{s} {\left| {\left( {{y_i} - {{\hat {y}}_i}} \right)} \right|}$$

In the above equation ENSE shows the error in NSE, ${y_i}$ presents the reference solutions and ${\hat {y}_i}$ indicates the proposed solutions, while *q*_1_ and *q*_3_ are the first and third quartiles.

## Simulations of the results

In this section, the simulation of the results for solving the P-II arising in nonlinear optics model using the MWNN-GA-IPA is presented. Findings from simulations with varying neuron counts and the implications for precision and convergence are additionally indicated as follows:

### Example I

Consider $\lambda = - 2$ and the numbers of neurons are taken as 10

20 $$\left\{ \begin{gathered} \frac{{{d^2}y(x)}}{{d{x^2}}}=2{y^3}(x)+xy(x) - 2, \hfill \\ y(0)=\frac{{dy(0)}}{{dx}}=0. \hfill \\ \end{gathered} \right.$$

A merit function for the above equation becomes as:

21 $${E_F}=\frac{1}{{10}}{\left( {\frac{{{d^2}}}{{dx^2}}{{\hat {y}}_i} - 2\hat {y}_{{_{i}}}^{3} - {x_i}{{\hat {y}}_i}+2} \right)^2}+\frac{1}{2}\left( {{{({{\hat {y}}_0})}^2}+\,{{\left( {{{\hat {y}^{\prime}}_0}} \right)}^2}} \right).$$

The numerical outcomes with the optimization of techniques MWNN-GA-IPA to optimize the above system (21) along with the performances of the outputs. These weights represent the parameters that fit all approaches most accurately and yield appropriate approximations of the results. In Example I, ten numbers of executions have been performed, which shows the computations for various neuronal counts to verify the suggested method for determining the best-fitting neurons. The MW is used as a merit function, which is capable to express the outcomes of various schemes as a series solution as:

22 $$\hat {y}(x)=\sum\limits_{{i=1}}^{{10}} {{k_i}\cos \left( {\frac{4}{3}({w_i}x+{j_i})} \right){e^{ - \frac{1}{2}{{({w_i}x+{j_i})}^2}}}}$$

The updated form of the Eq. (22) becomes as:

23 $$\begin{gathered} \hat {y}(x)=0.4618\cos \left( {\frac{4}{3}( - 3.6323x - 7.0594)} \right){e^{ - \frac{1}{2}{{( - 3.6323x - 7.0594)}^2}}} \hfill \\ \,\,\,\,\,\,\,\,\,\,\, - 0.0036\cos \left( {\frac{4}{3}(8.4115x+1.8574)} \right){e^{ - \frac{1}{2}{{(8.4115x+1.8574)}^2}}} \hfill \\ \,\,\,\,\,\,\,\,\,\,\,+0.3203\cos \left( {\frac{4}{3}(2.4373x - 0.1534)} \right){e^{ - \frac{1}{2}{{(2.4373x - 0.1534)}^2}}} \hfill \\ \,+...\, - 0.1046\cos \left( {\frac{4}{3}( - 0.3486x+2.4463)} \right){e^{ - \frac{1}{2}{{( - 0.3486x+2.4463)}^2}}}. \hfill \\ \end{gathered}$$

**Fig. 2:**
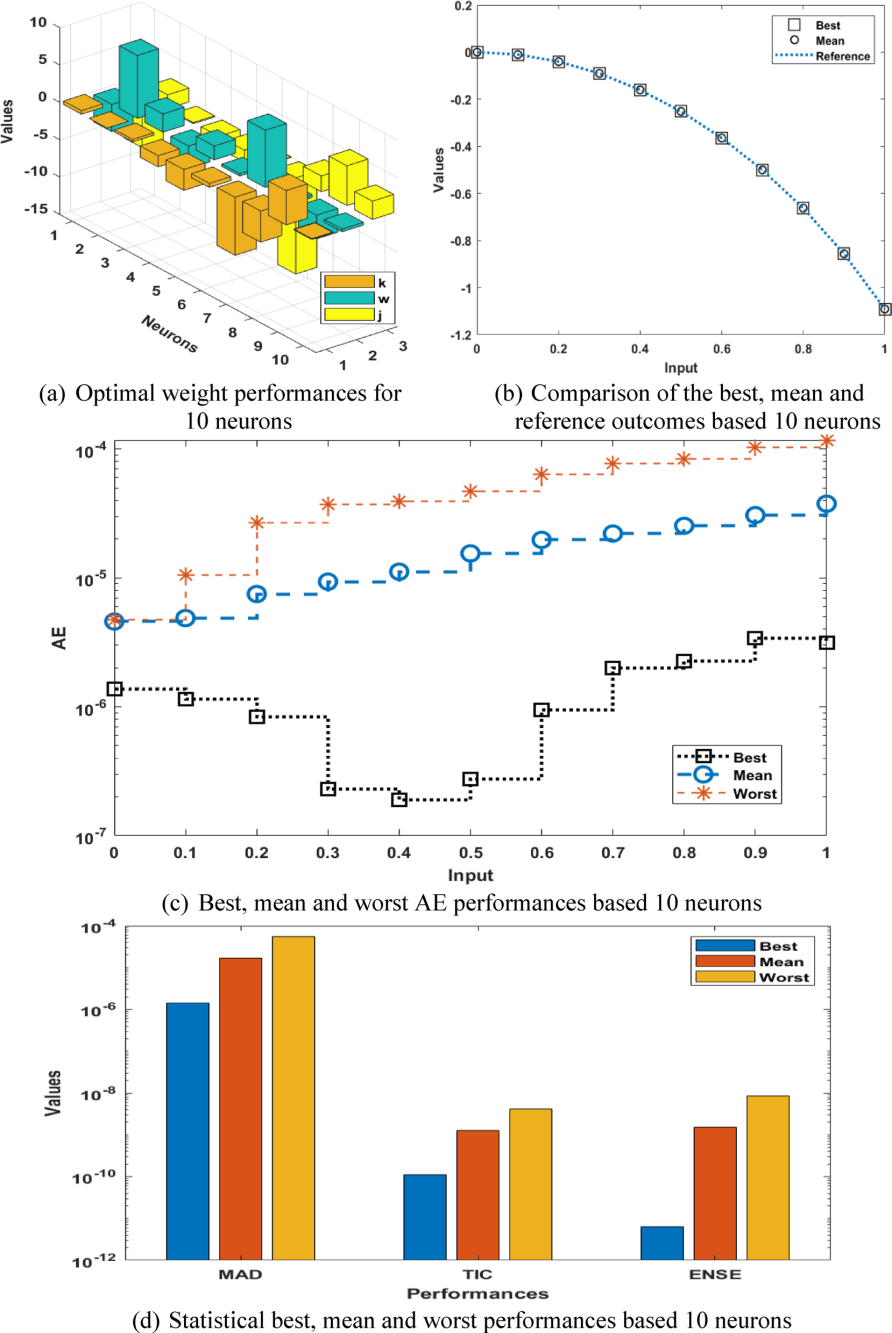
Best weights, comparison of the results, AE performances and statistical results for the P-II arising in nonlinear optics model.

The optimal weight vectors, comparison of the results, absolute error (AE) performances and statistical results for the P-II arising in nonlinear optics model are accessible in Fig. 2. The best weights have been mentioned in the Fig. 2(a) based on 10 runs. The overlapping of the best and mean results in comparison with the reference results is given in Fig. 2(b). It is observed that the mean and best solutions in ten runs has overlapped with the reference solutions, which shows the capability of the proposed technique for presenting the solutions of the P-II arising in nonlinear optics model. The AE values based on the best, mean and even worst results are presented in Fig. 2(c). It is seen that the best, mean and worst result performances are found around 10^−06^ to 10^−07^, 10^−05^ to 10^−06^, and 10^−04^ to 10^−05^. AE presents a quantitative ration of the difference between the reference and proposed values, which is mathematically defined as: ${\text{AE=}}\left| {\left( {{y_i} - {{\hat {y}}_i}} \right)} \right|$.

These small or negligible AE presents the correctness of the proposed MWNN-GA-IPA technique for presenting the solutions of the P-II arising in nonlinear optics model. The statistical best, mean and worst performances based 10 numbers of neurons are presented in Fig. 2(d). Three tests based on the MAD, TIC and ENSE are presented. It is noticed that the best, mean and worst MAD values are performed close to 10^−06^, 10^−05^, and 10^−04^. The best, mean and worst TIC values are performed close to 10^–10^, 10^−09^, and 10^−08^. The best, mean and worst EVAF performances are noticed, which are close to 10^–11^, 10^−09^, and 10^−08^. These Statistical tests provide the support in order to assess the neural network performance. By using the comparison performances of the reference and proposed results, statistical tests represent the insights into the system’s exactness, accuracy, and consistency.

**Fig. 3:**
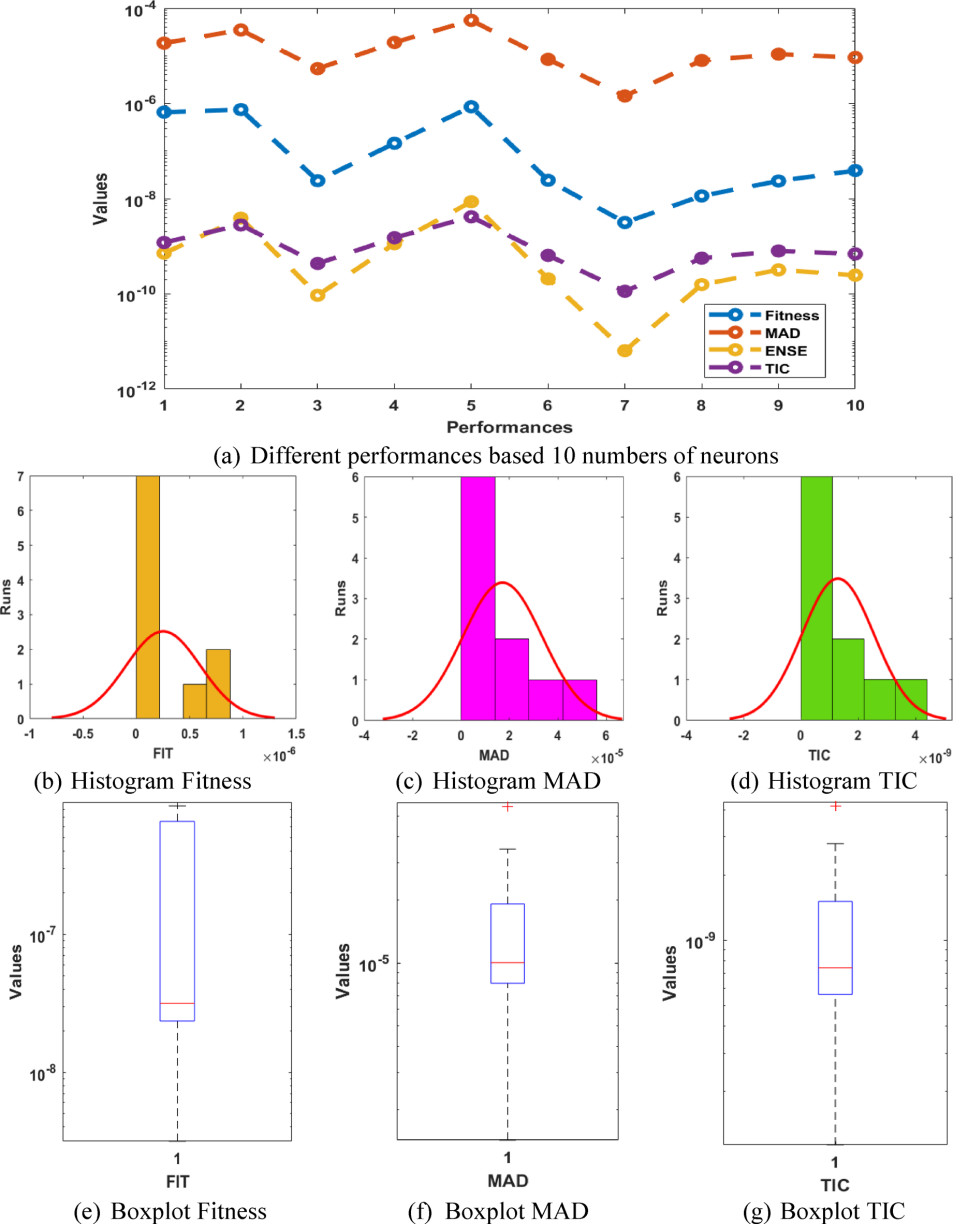
Different test performances, histogram and boxplot for the P-II arising in nonlinear optics system.

The statistical performances, histogram and boxplot for the P-II arising in nonlinear optics model are presented in Fig. 3. It is shown that most of the values based on the Fitness, MAD, TIC and ENSE are performed into reasonable percentages, which shows the reliability of the proposed scheme for solving the model.

Table 2 presents the statistical operator performances based on minimum (Min), mean, Med, SD and SIR. These negligible values enhance the reliability of the proposed scheme.

**Table 2:** Statistical measures based MWNN-GA-IPA for the P-II arising in nonlinear optics model.

Mode	$\hat {y}(x)$										
	0	0.1	0.2	0.3	0.4	0.5	0.6	0.7	0.8	0.9	1
Min	1 × 10^−7^	1 × 10^−7^	8 × 10^−7^	2 × 10^−7^	1 × 10^−7^	2 × 10^−7^	9 × 10^−7^	2 × 10^−6^	2 × 10^−6^	3 × 10^−6^	3 × 10^−6^
Mean	1 × 10^−5^	1 × 10^−5^	2 × 10^−5^	3 × 10^−5^	4 × 10^−5^	4 × 10^−5^	6 × 10^−5^	7 × 10^−5^	8 × 10^−5^	1 × 10^−4^	1 × 10^−4^
SD	1 × 10^−6^	2 × 10^−6^	4 × 10^−6^	6 × 10^−6^	7 × 10^−6^	9 × 10^−6^	1 × 10^−5^	1 × 10^−5^	1 × 10^−5^	1 × 10^−5^	2 × 10^−5^
Med	4 × 10^−6^	4 × 10^−6^	7 × 10^−6^	9 × 10^−6^	1 × 10^−5^	1 × 10^−5^	2 × 10^−5^	2 × 10^−5^	2 × 10^−5^	3 × 10^−5^	3 × 10^−5^
SIR	2 × 10^−6^	4 × 10^−6^	1 × 10^−6^	1 × 10^−6^	3 × 10^−6^	6 × 10^−6^	8 × 10^−6^	7 × 10^−6^	9 × 10^−6^	1 × 10^−5^	1 × 10^−5^

### Example II

Consider $\lambda = - 2$ and the numbers of neurons are taken as 15.

24 $$\left\{ \begin{gathered} \frac{{{d^2}y(x)}}{{d{x^2}}}=2{y^3}(x)+xy(x) - 2, \hfill \\ y(0)=\frac{{dy(0)}}{{dx}}=0. \hfill \\ \end{gathered} \right.$$

A merit function for Eq. (24) is given as:

25 $${E_F}=\frac{1}{{15}}{\left( {\frac{{{d^2}}}{{dx^2}}{{\hat {y}}_i} - 2\hat {y}_{{_{i}}}^{3} - {x_i}{{\hat {y}}_i}+2} \right)^2}+\frac{1}{2}\left( {{{({{\hat {y}}_0})}^2}+\,{{\left( {{{\hat {y}^{\prime}}_0}} \right)}^2}} \right).$$

The numerical outcomes with the optimization of MWNN-GA-IPA to optimize the above system (25) and the performances of the outputs are presented in Eq. (27). These weights represent the parameters that fit all approaches most accurately and yield appropriate calculations of the results:

26 $$\hat {y}(x)=\sum\limits_{{i=1}}^{{15}} {{k_i}\cos \left( {\frac{4}{3}({w_i}x+{j_i})} \right){e^{ - \frac{1}{2}{{({w_i}x+{j_i})}^2}}}} .$$

The updated form of the Eq. (26) becomes as:

27 $$\begin{gathered} \hat {y}(x)=3.2651\cos \left( {\frac{4}{3}( - 1.2013x - 7.7897)} \right){e^{ - \frac{1}{2}{{( - 1.2013x - 7.7897)}^2}}} \hfill \\ \,\,\,\,\,\,\,\,\,\,\,+4.4005\cos \left( {\frac{4}{3}( - 1.7321x+2.0080)} \right){e^{ - \frac{1}{2}{{( - 1.7321x+2.0080)}^2}}} \hfill \\ \,\,\,\,\,\,\,\,\,\,\, - 2.6060\cos \left( {\frac{4}{3}( - 4.6673x+11.2421)} \right){e^{ - \frac{1}{2}{{( - 4.6673x+11.2421)}^2}}} \hfill \\ \,+....\, - 4.1788\cos \left( {\frac{4}{3}( - 0.2409x+1.3499)} \right){e^{ - \frac{1}{2}{{( - 0.2409x+1.3499)}^2}}} \hfill \\ \end{gathered}$$

**Fig. 4:**
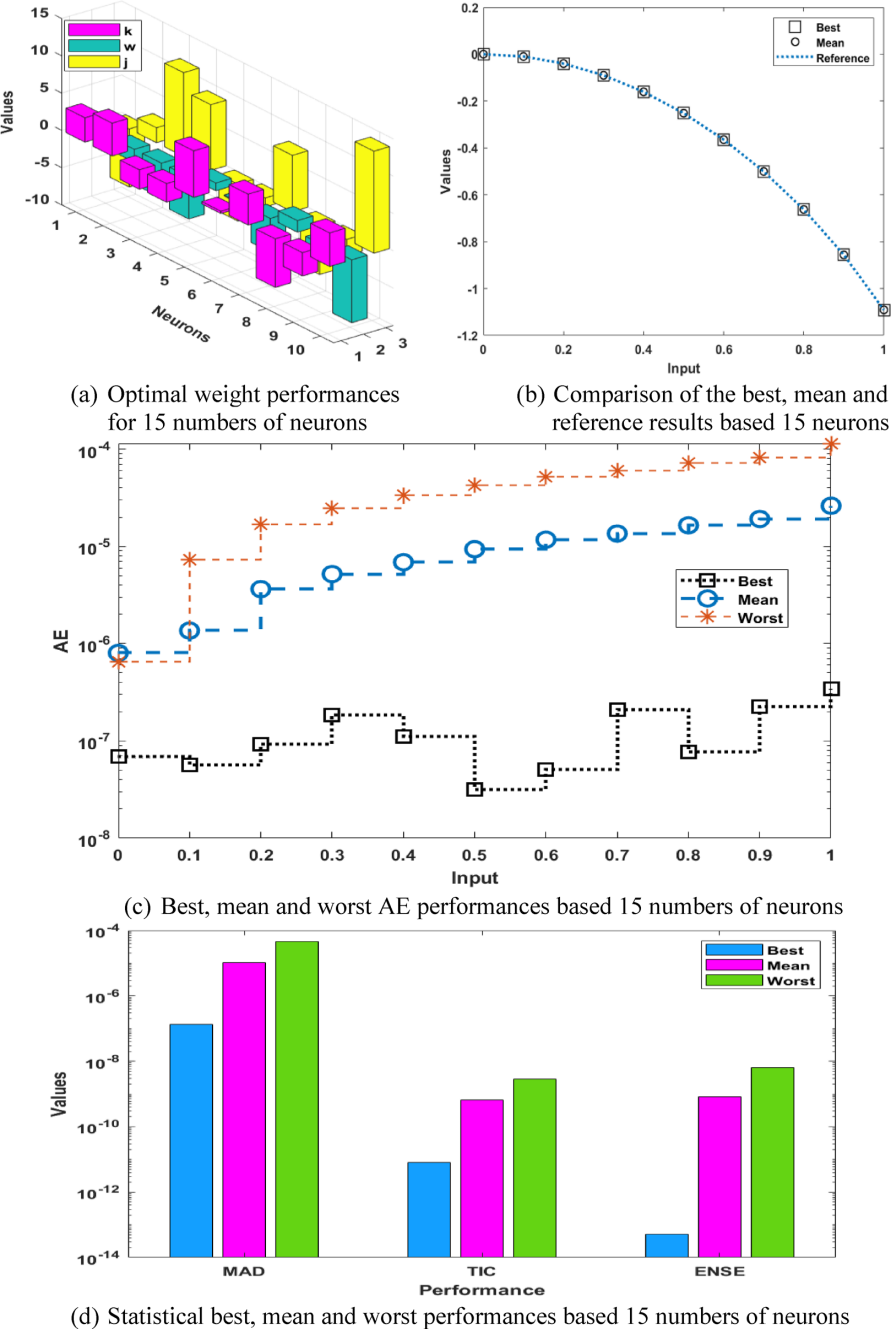
Best weights, comparison of the results, AE performances and statistical results for the P-II arising in nonlinear optics model using 15 numbers of neurons.

**Fig. 5:**
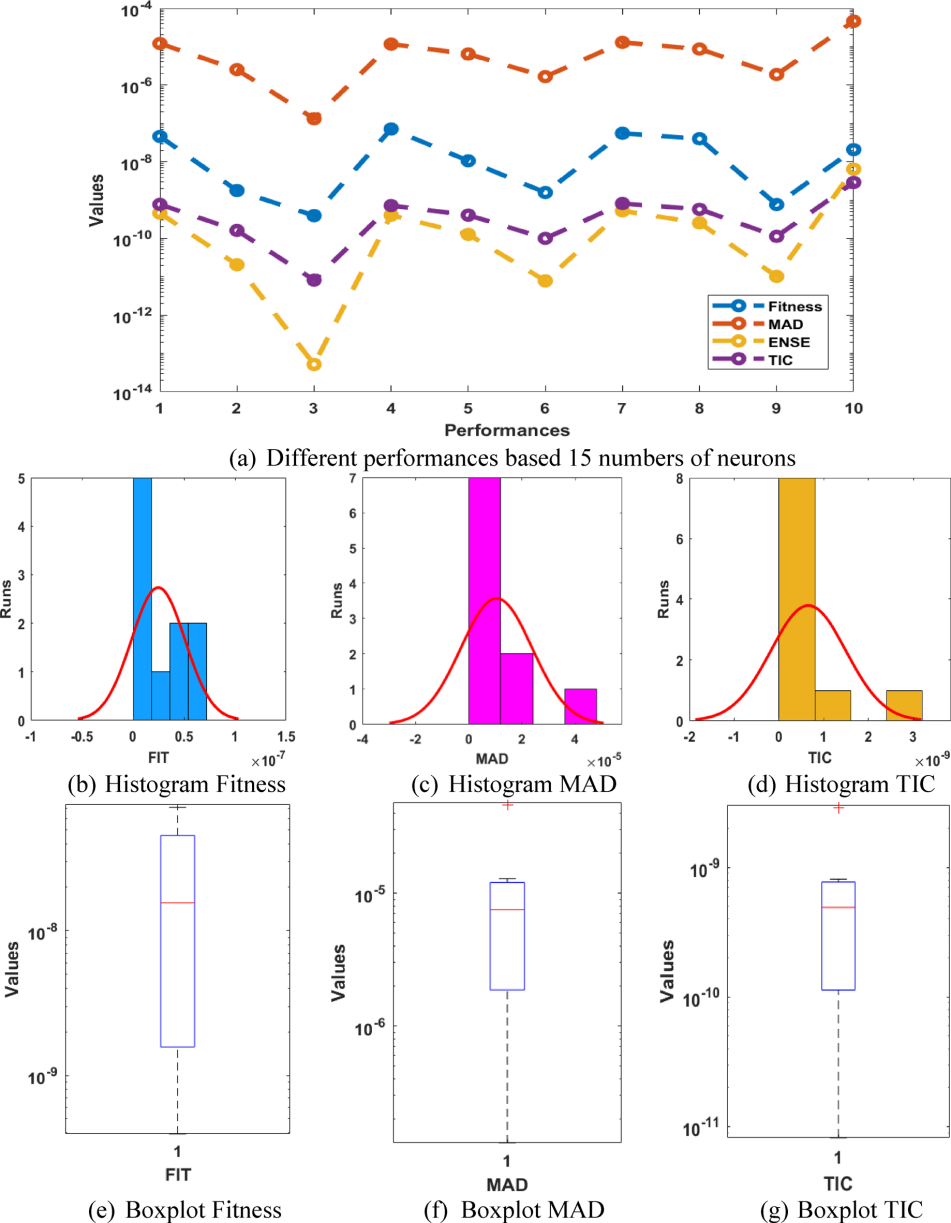
Different test performances, histogram and boxplot for the P-II arising in nonlinear optics model using 15 numbers of neurons.

The optimal weights, result comparison, AE performances and statistical results for the P-II arising in nonlinear optics model are accessible in Fig. 4. The best weight vectors have been mentioned in the Fig. 4(a) based on 15 runs. The overlapping of the best and mean results in comparison with the reference outcomes is provided in Fig. 4(b). It is observed that the mean and best solutions in ten runs has overlapped with the reference solutions, which shows the ability of the proposed technique for presenting the solutions of the P-II arising in nonlinear optics model. The AE values based on the best, mean and even worst results are presented in Fig. 4(c). It is seen that the best, mean and worst result performances are found around 10^−07^ to 10^−08^, 10^−06^ to 10^−07^, and 10^−04^ to 10^−05^. These AE based on 15 neurons perform slightly better as compared to ten numbers of neurons. The statistical best, mean and worst performances based 15 numbers of neurons are presented in Fig. 4(d). The tests based on the MAD, TIC and ENSE performances are also performed slight better as compared to 10 numbers of neuron. The similar depiction has been reported in Fig. 5, which shows more reliable solutions in comparison with 10 numbers of neurons. On the basis of these results, one can claim that the reliability and consistency is slightly better for 15 neurons instead of 10 numbers of neurons.

Table 3 shows the statistical operator Min, mean, Med, SD and SIR performances. These values are also reported slightly better in comparison with the 10 numbers of neurons.

**Table 3:** Statistical measures based MWNN-GA-IPA for the P-II arising in nonlinear optics model.

Mode	$\hat {y}(x)$										
	0	0.1	0.2	0.3	0.4	0.5	0.6	0.7	0.8	0.9	1
Min	2 × 10^−8^	5 × 10^−8^	9 × 10^−8^	1 × 10^−7^	1 × 10^−7^	3 × 10^−8^	5 × 10^−8^	2 × 10^−7^	7 × 10^−8^	2 × 10^−7^	3 × 10^−7^
Mean	3 × 10^−6^	7 × 10^−6^	1 × 10^−5^	2 × 10^−5^	3 × 10^−5^	4 × 10^−5^	5 × 10^−5^	6 × 10^−5^	7 × 10^−5^	8 × 10^−5^	1 × 10^−4^
SD	5 × 10^−7^	5 × 10^−7^	1 × 10^−6^	2 × 10^−6^	3 × 10^−6^	6 × 10^−6^	8 × 10^−6^	9 × 10^−6^	1 × 10^−5^	1 × 10^−5^	2 × 10^−5^
Med	8 × 10^−7^	1 × 10^−6^	3 × 10^−6^	5 × 10^−6^	6 × 10^−6^	9 × 10^−6^	1 × 10^−5^	1 × 10^−5^	1 × 10^−5^	1 × 10^−5^	2 × 10^−5^
SIR	3 × 10^−7^	3 × 10^−7^	2 × 10^−6^	2 × 10^−6^	3 × 10^−6^	4 × 10^−6^	5 × 10^−6^	6 × 10^−6^	8 × 10^−6^	9 × 10^−6^	1 × 10^−5^

### Example III

Consider $\lambda = - 2$ and the numbers of neurons are taken as 30

28 $$\left\{ \begin{gathered} \frac{{{d^2}y(x)}}{{d{x^2}}}=2{y^3}(x)+xy(x) - 2, \hfill \\ y(0)=\frac{{dy(0)}}{{dx}}=0. \hfill \\ \end{gathered} \right.$$

A merit function for Eq. (28) is given as:

29 $${E_F}=\frac{1}{{30}}{\left( {\frac{{{d^2}}}{{dx^2}}{{\hat {y}}_i} - 2\hat {y}_{{_{i}}}^{3} - {x_i}{{\hat {y}}_i}+2} \right)^2}+\frac{1}{2}\left( {{{({{\hat {y}}_0})}^2}+\,{{\left( {{{\hat {y}^{\prime}}_0}} \right)}^2}} \right).$$

The numerical outcomes with the optimization of techniques MWNN-GA-IPA to optimize the above system (29) and the performances of the outputs have been presented in Eq. (31). These weights represent the parameters that fit all approaches most accurately and yield appropriate approximations of the results.

30 $$\hat {y}(x)=\sum\limits_{{i=1}}^{{30}} {{k_i}\cos \left( {\frac{4}{3}({w_i}x+{j_i})} \right){e^{ - \frac{1}{2}{{({w_i}x+{j_i})}^2}}}}$$

The updated form of the Eq. (30) becomes as:

31 $$\begin{gathered} \hat {y}(x)= - 1.8246\cos \left( {\frac{4}{3}(0.6583x+1.2763)} \right){e^{ - \frac{1}{2}{{(0.6583x+1.2763)}^2}}} \hfill \\ \,\,\,\,\,\,\,\,\,\,\,\,\,\,\, - 0.9461\cos \left( {\frac{4}{3}(0.8585x - 0.1698)} \right){e^{ - \frac{1}{2}{{(0.8585x - 0.1698)}^2}}} \hfill \\ \,\,\,\,\,\,\,\,\,\,\,\,\,\,\, - 4.7865\cos \left( {\frac{4}{3}(0.7008x - 4.5199)} \right){e^{ - \frac{1}{2}{{(0.7008x - 4.5199)}^2}}} \hfill \\ \,\,\,\,\,\,\,\,\,\,\,\,\,\,\, - 0.3754\cos \left( {\frac{4}{3}( - 1.2383x+0.9086)} \right){e^{ - \frac{1}{2}{{( - 1.2383x+0.9086)}^2}}} \hfill \\ \,\,\,\,\,+....\,+0.7208\cos \left( {\frac{4}{3}( - 2.1385x - 1.9510)} \right){e^{ - \frac{1}{2}{{( - 2.1385x - 1.9510)}^2}}} \hfill \\ \end{gathered}$$

**Fig. 6:**
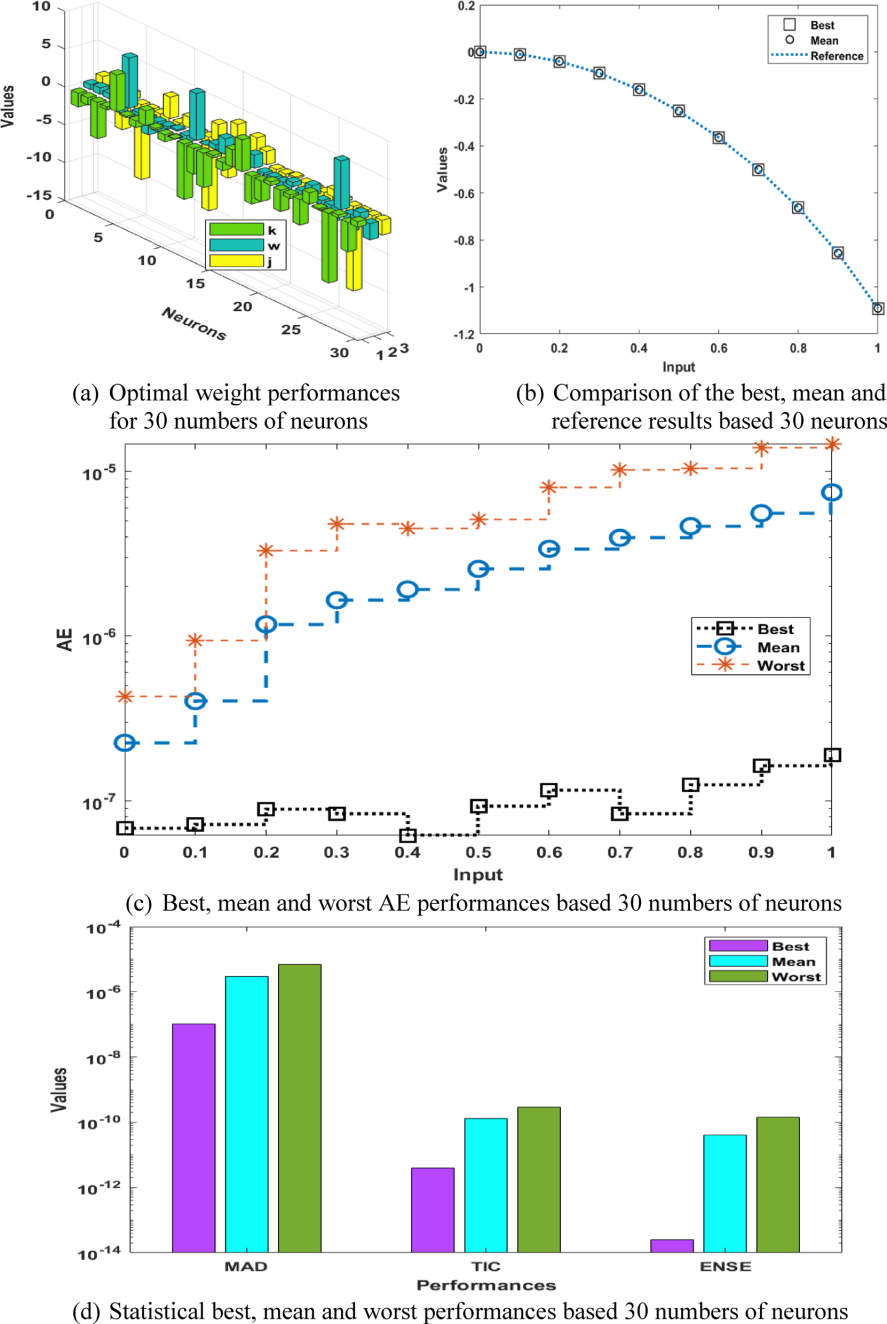
Best weights, comparison of the results, AE performances and statistical results for the P-II arising in nonlinear optics model using 30 numbers of neurons.

**Fig. 7:**
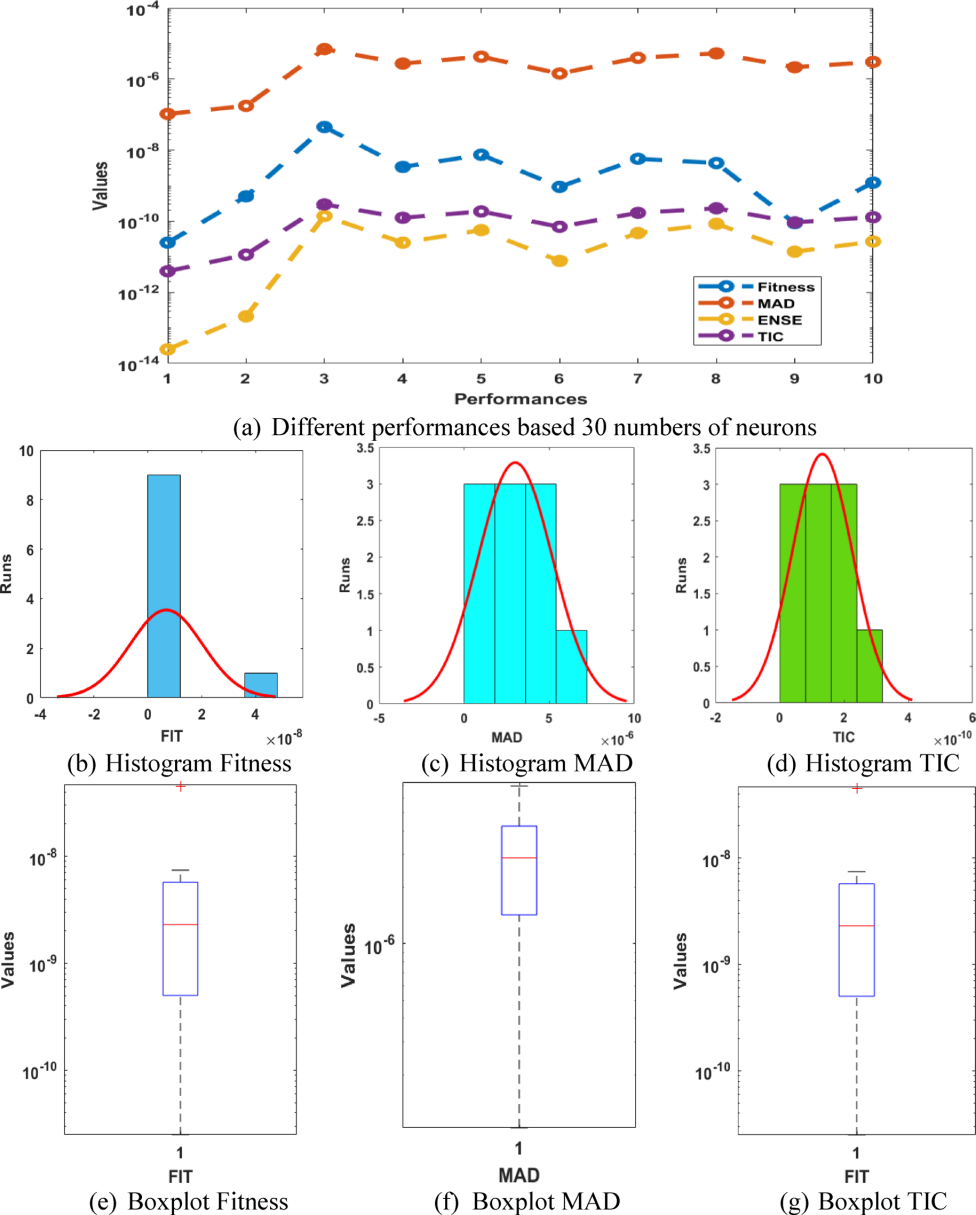
Different test performances, histogram and boxplot for the P-II arising in nonlinear optics model using 30 numbers of neurons.

The optimal weight vectors, comparison of the results, AE performances and statistical results for the P-II arising in nonlinear optics model are presented in Fig. 6, while ten numbers of executions using different tests are provided in Fig. 7. On the behalf of these results, it is observed that the preciseness and reliability is obtained better for 30 numbers of neurons instead of 10 and 15. One can conclude that by increasing numbers of neurons, the performances of the solver are getting better for solving the model. Table 4 provides the results on the basis of 30 numbers of neurons, which shows more precise values as compared to 10 and 15 numbers of neurons for solving the model.

**Table 4:** Statistical measures based MWNN-GA-IPA for the P-II arising in nonlinear optics model.

Mode	$\hat {y}(x)$										
	0	0.1	0.2	0.3	0.4	0.5	0.6	0.7	0.8	0.9	1
Min	1 × 10^−8^	7 × 10^−9^	8 × 10^−8^	8 × 10^−8^	2 × 10^−8^	9 × 10^−8^	1 × 10^−7^	7 × 10^−8^	1 × 10^−7^	6 × 10^−8^	1 × 10^−7^
Mean	8 × 10^−7^	9 × 10^−7^	3 × 10^−6^	4 × 10^−6^	4 × 10^−6^	5 × 10^−6^	8 × 10^−6^	1 × 10^−5^	1 × 10^−5^	1 × 10^−5^	1 × 10^−5^
SD	1 × 10^−7^	4 × 10^−7^	1 × 10^−6^	1 × 10^−6^	1 × 10^−6^	2 × 10^−6^	3 × 10^−6^	3 × 10^−6^	4 × 10^−6^	5 × 10^−6^	7 × 10^−6^
Med	2 × 10^−7^	4 × 10^−7^	1 × 10^−6^	1 × 10^−6^	1 × 10^−6^	2 × 10^−6^	3 × 10^−6^	4 × 10^−6^	4 × 10^−6^	5 × 10^−6^	7 × 10^−6^
SIR	9 × 10^−8^	3 × 10^−7^	5 × 10^−7^	7 × 10^−7^	9 × 10^−7^	1 × 10^−6^	1 × 10^−6^	2 × 10^−6^	2 × 10^−6^	3 × 10^−6^	2 × 10^−6^

### Metrics for convergence analysis

The convergence analysis based on different matrices is shown as:

#### NSE

- It processes the system’s projecting power, which has a range of -*∞* to 1.
- The performances around 1 represent good value.
- Measure NSE based on predicted and observed performances.

#### TIC

- It processes the correctness of system’s prediction.
- Performances 0 to 1 shows the less values representing improved presentation.
- Calculate TIC based on predicted and observed performances.

#### SIR

- It calculates the robustness and inconsistency of system.
- Perform SIR based on the interquartile range of the anticipated performance.

#### MAD

- It performs the average modification in predicted and observed performances.
- Lower values of MAD represent better performance of system.

For the convergence analysis, the matrices NSE, TIC, SIR, and MAD have been monitored based on the simulation or training. The metrics are visualized over generation or time to measure convergence. The metric performances converge or stabilize to a fixed state, which presents the convergence of system. Error bounds (EBs) denotes to the ranges or limits in which the real error in actual and predicted performances is predictable. EBs indicate an assessable amount of the minimum or maximum error amount, which may exist in the prediction of system. EBs have been performed based on AE, or other metrics. It is widely applied to assess the reliability and performance of systems, and approximation schemes.

## Conclusion

In this study, a novel design of MWNN is presented for the numerical outputs of the P-II arising in nonlinear optics. The mathematical P-II arising in nonlinear optics form is one of the nonlinear model, which is always difficult to solve by using the traditional schemes. Some conclusions of this research are provided as:

- The results of the P-II arising in nonlinear optics have been successfully presented by using the proposed MWNN.
- An error based merit function has been designed using the sense of differential model and its initial or boundary conditions.
- The optimization is performed by using the hybrid computing terminologies of the global and local search schemes.
- The global search GA and local search IPA is used to solve the model.
- The correctness of the designed procedure has been observed through the overlapping of the proposed and reference results.
- The negligible AE around 10^−05^ to 10^−07^ provide the accurateness of the designed solver.
- The statistical analysis based on the multiple independent trials has also been performed to check the reliability of the proposed MWNN-GA-IPA in order to P-II arising in nonlinear optics.
- It is observed through different tests that the maximum numbers of neurons provide more accurate results in comparison with the small numbers of neurons.

In future, the proposed MWNN-GA-IPA can be used to solve various problems including magnetohydrodynamic stagnation point Ree-Eyring flow model^32^, convective heat transfer through straight fin model^33^, thermal enhancement in the Ternary hybrid nanofluid model^34^, dengue transmission system^35^, Chikungunya virus model^36^, and corneal geometry of the human eye model^37^.

## Appendix

The equation based on A.1, A.2 and A.3 for 10, 15 and 30 numbers of neurons are presented.

A.1 $$\begin{gathered} \hat {y}(x)=0.4618\cos \left( {\frac{4}{3}( - 3.6323x - 7.0594)} \right){e^{ - \frac{1}{2}{{( - 3.6323x - 7.0594)}^2}}} \hfill \\ \,\,\,\,\,\,\,\,\,\,\, - 0.0036\cos \left( {\frac{4}{3}(8.4115x+1.8574)} \right){e^{ - \frac{1}{2}{{(8.4115x+1.8574)}^2}}} \hfill \\ \,\,\,\,\,\,\,\,\,\,\,+0.3203\cos \left( {\frac{4}{3}(2.4373x - 0.1534)} \right){e^{ - \frac{1}{2}{{(2.4373x - 0.1534)}^2}}} \hfill \\ \,\,\,\,\,\,\,\,\,\,\, - 1.5075\cos \left( {\frac{4}{3}( - 3.0204x - 2.1753)} \right){e^{ - \frac{1}{2}{{( - 3.0204x - 2.1753)}^2}}} \hfill \\ \,\,\,\,\,\,\,\,\,\,\, - 2.2893\cos \left( {\frac{4}{3}(1.8432x - 1.5826)} \right){e^{ - \frac{1}{2}{{(1.8432x - 1.5826)}^2}}} \hfill \\ \,\,\,\,\,\,\,\,\,\,\, - 0.5308\cos \left( {\frac{4}{3}( - 0.3169x+0.0508)} \right){e^{ - \frac{1}{2}{{( - 0.3169x+0.0508)}^2}}} \hfill \\ \,\,\,\,\,\,\,\,\,\,\, - 7.7760\cos \left( {\frac{4}{3}(7.6750x - 12.8669)} \right){e^{ - \frac{1}{2}{{(7.6750x - 12.8669)}^2}}} \hfill \\ \,\,\,\,\,\,\,\,\,\,\, - 4.1702\cos \left( {\frac{4}{3}( - 0.3610x+2.1018)} \right){e^{ - \frac{1}{2}{{( - 0.3610x+2.1018)}^2}}} \hfill \\ \,\,\,\,\,\,\,\,\,\,\,+4.5906\cos \left( {\frac{4}{3}( - 3.1549x+5.3240)} \right){e^{ - \frac{1}{2}{{( - 3.1549x+5.3240)}^2}}} \hfill \\ \,\,\,\,\,\,\,\,\,\,\, - 0.1046\cos \left( {\frac{4}{3}( - 0.3486x+2.4463)} \right){e^{ - \frac{1}{2}{{( - 0.3486x+2.4463)}^2}}} \hfill \\ \end{gathered}$$

A.2 $$\begin{gathered} \hat {y}(x)=3.2651\cos \left( {\frac{4}{3}( - 1.2013x - 7.7897)} \right){e^{ - \frac{1}{2}{{( - 1.2013x - 7.7897)}^2}}} \hfill \\ \,\,\,\,\,\,\,\,\,\,\,+4.4005\cos \left( {\frac{4}{3}( - 1.7321x+2.0080)} \right){e^{ - \frac{1}{2}{{( - 1.7321x+2.0080)}^2}}} \hfill \\ \,\,\,\,\,\,\,\,\,\,\, - 2.6060\cos \left( {\frac{4}{3}( - 4.6673x+11.2421)} \right){e^{ - \frac{1}{2}{{( - 4.6673x+11.2421)}^2}}} \hfill \\ \,\,\,\,\,\,\,\,\,\,\, - 2.4982\cos \left( {\frac{4}{3}( - 5.6320x+8.8015)} \right){e^{ - \frac{1}{2}{{( - 5.6320x+8.8015)}^2}}} \hfill \\ \,\,\,\,\,\,\,\,\,\,\,+\,6.2413\cos \left( {\frac{4}{3}(1.0392x - 4.9653)} \right){e^{ - \frac{1}{2}{{(1.0392x - 4.9653)}^2}}} \hfill \\ \,\,\,\,\,\,\,\,\,\,\, - 0.3071\cos \left( {\frac{4}{3}( - 0.1209x - 1.2032)} \right){e^{ - \frac{1}{2}{{( - 0.1209x - 1.2032)}^2}}} \hfill \\ \,\,\,\,\,\,\,\,\,\,\,+\,4.1385\cos \left( {\frac{4}{3}( - 4.5148x+7.5654)} \right){e^{ - \frac{1}{2}{{( - 4.5148x+7.5654)}^2}}} \hfill \\ \,\,\,\,\,\,\,\,\,\,\, - 6.5192\cos \left( {\frac{4}{3}(1.5564x - 6.5634)} \right){e^{ - \frac{1}{2}{{(1.5564x - 6.5634)}^2}}} \hfill \\ \,\,\,\,\,\,\,\,\,\,\, - 3.1314\cos \left( {\frac{4}{3}( - 2.0487x - 2.1745)} \right){e^{ - \frac{1}{2}{{( - 2.0487x - 2.1745)}^2}}} \hfill \\ \,\,\,\,\,\,\,\,\,\,\,+4.5010\cos \left( {\frac{4}{3}( - 8.4327x+13.6767)} \right){e^{ - \frac{1}{2}{{( - 8.4327x+13.6767)}^2}}} \hfill \\ \,\,\,\,\,\,\,\,\,\,\,+1.0470\cos \left( {\frac{4}{3}( - 0.4835x+8.7932)} \right){e^{ - \frac{1}{2}{{( - 0.4835x+8.7932)}^2}}} \hfill \\ \,\,\,\,\,\,\,\,\,\,\, - 6.6761\cos \left( {\frac{4}{3}(4.4547x - 8.1302)} \right){e^{ - \frac{1}{2}{{(4.4547x - 8.1302)}^2}}} \hfill \\ \,\,\,\,\,\,\,\,\,\, - 4.1717\cos \left( {\frac{4}{3}( - 0.0140x - 1.2020)} \right){e^{ - \frac{1}{2}{{( - 0.0140x - 1.2020)}^2}}} \hfill \\ \,\,\,\,\,\,\,\,\,\, - 3.8258\cos \left( {\frac{4}{3}( - 2.4908x+6.5464)} \right){e^{ - \frac{1}{2}{{( - 2.4908x+6.5464)}^2}}} \hfill \\ \,\,\,\,\,\,\,\,\,\, - 4.1788\cos \left( {\frac{4}{3}( - 0.2409x+1.3499)} \right){e^{ - \frac{1}{2}{{( - 0.2409x+1.3499)}^2}}} \hfill \\ \end{gathered}$$

A.3 $$\begin{gathered} \hat {y}(x)= - 1.82\cos \left( {\frac{4}{3}(0.6583x+1.2763)} \right){e^{ - \frac{1}{2}{{(0.6583x+1.2763)}^2}}} \hfill \\ \, - 0.94\cos \left( {\frac{4}{3}(0.85x - 0.16)} \right){e^{ - \frac{1}{2}{{(0.85x - 0.16)}^2}}} - 4.78\cos \left( {\frac{4}{3}(0.7x - 4.51)} \right){e^{ - \frac{1}{2}{{(0.70x - 4.51)}^2}}} \hfill \\ - 0.37\cos \left( {\frac{4}{3}( - 1.23x+0.90)} \right){e^{ - \frac{1}{2}{{( - 1.23x+0.9)}^2}}} - 4.83\cos \left( {\frac{4}{3}(6.6x - 9.89)} \right){e^{ - \frac{1}{2}{{(6.6x - 9.89)}^2}}} \hfill \\ - 0.18\cos \left( {\frac{4}{3}( - 0.54x - 1.13)} \right){e^{ - \frac{1}{2}{{( - 0.54x - 1.13)}^2}}} - 0.88\cos \left( {\frac{4}{3}( - 2.3x - 1.9)} \right){e^{ - \frac{1}{2}{{( - 2.3x - 1.9)}^2}}} \hfill \\ +1.94\cos \left( {\frac{4}{3}( - 1.29x+2.83)} \right){e^{ - \frac{1}{2}{{( - 1.29x+2.83)}^2}}}+0.22\cos \left( {\frac{4}{3}( - 0.3x - 0.5)} \right){e^{ - \frac{1}{2}{{( - 0.3x - 0.53)}^2}}} \hfill \\ - 0.92\cos \left( {\frac{4}{3}(0.18x+1.386)} \right){e^{ - \frac{1}{2}{{(0.18x+1.38)}^2}}}\,+0.07\cos \left( {\frac{4}{3}(0.85x - 0.76)} \right){e^{ - \frac{1}{2}{{(0.85x - 0.76)}^2}}} \hfill \\ - 7.31\cos \left( {\frac{4}{3}(6.20x - 9.72)} \right){e^{ - \frac{1}{2}{{(6.20x - 9.72)}^2}}}\, - 3.38\cos \left( {\frac{4}{3}( - 0.03x+2.2)} \right){e^{ - \frac{1}{2}{{( - 0.03x+2.27)}^2}}} \hfill \\ - 4.54\cos \left( {\frac{4}{3}(0.6x - 4.20)} \right){e^{ - \frac{1}{2}{{(0.66x - 4.20)}^2}}}\,+0.33\cos \left( {\frac{4}{3}(2.06x+3.51)} \right){e^{ - \frac{1}{2}{{(2.06x+3.51)}^2}}} \hfill \\ - 1.07\cos \left( {\frac{4}{3}(0.05x - 1.45)} \right){e^{ - \frac{1}{2}{{(0.05x - 1.45)}^2}}}\,+2.28\cos \left( {\frac{4}{3}( - 0.6x+2.9)} \right){e^{ - \frac{1}{2}{{( - 0.679x+2.952)}^2}}} \hfill \\ +4.21\cos \left( {\frac{4}{3}(1.73x+1.77)} \right){e^{ - \frac{1}{2}{{(1.73x+1.77)}^2}}} - 3.64\cos \left( {\frac{4}{3}( - 0.32x - 1.4)} \right){e^{ - \frac{1}{2}{{( - 0.32x - 1.40)}^2}}} \hfill \\ - 2.71\cos \left( {\frac{4}{3}(0.82x - 0.44)} \right){e^{ - \frac{1}{2}{{(0.82x - 0.44)}^2}}}\,+0.48\cos \left( {\frac{4}{3}( - 1.50x+0.2)} \right){e^{ - \frac{1}{2}{{( - 1.50x+0.20)}^2}}} \hfill \\ - 2.81\cos \left( {\frac{4}{3}(0.55x - 1.16)} \right){e^{ - \frac{1}{2}{{(0.55x - 1.16)}^2}}}\, - 0.50\cos \left( {\frac{4}{3}( - 1.09x - 1.32)} \right){e^{ - \frac{1}{2}{{( - 1.09x - 1.32)}^2}}} \hfill \\ - 3.38\cos \left( {\frac{4}{3}(1.35x - 2.65)} \right){e^{ - \frac{1}{2}{{(1.35x - 2.65)}^2}}}\,+1.48\cos \left( {\frac{4}{3}( - 0.61x+0.96)} \right){e^{ - \frac{1}{2}{{( - 0.61x+0.96)}^2}}} \hfill \\ - 0.20\cos \left( {\frac{4}{3}( - 2.2x+0.35)} \right){e^{ - \frac{1}{2}{{( - 2.25x+0.35)}^2}}} - 9.2\cos \left( {\frac{4}{3}(6.468x - 11.1)} \right){e^{ - \frac{1}{2}{{(6.46x - 11.17)}^2}}} \hfill \\ +0.39\cos \left( {\frac{4}{3}( - 2.3x - 1.54)} \right){e^{ - \frac{1}{2}{{( - 2.3x - 1.54)}^2}}}\,\, - 3.87\cos \left( {\frac{4}{3}(0.83x - 2.68)} \right){e^{ - \frac{1}{2}{{(0.83x - 2.68)}^2}}} \hfill \\ \,+0.7208\cos \left( {\frac{4}{3}( - 2.1385x - 1.9510)} \right){e^{ - \frac{1}{2}{{( - 2.1385x - 1.9510)}^2}}} \hfill \\ \end{gathered}$$.

## Data Availability

The datasets used and/or analyzed during the current study are available from the corresponding author on reasonable request.
